# Mitophagy Protects the Retina Against Anti-Vascular Endothelial Growth Factor Therapy-Driven Hypoxia *via* Hypoxia-Inducible Factor-1α Signaling

**DOI:** 10.3389/fcell.2021.727822

**Published:** 2021-11-01

**Authors:** Yimeng Sun, Feng Wen, Chun Yan, Lishi Su, Jiawen Luo, Wei Chi, Shaochong Zhang

**Affiliations:** ^1^State Key Laboratory of Ophthalmology, Zhongshan Ophthalmic Center, Sun Yat-sen University, Guangzhou, China; ^2^Shenzhen Key Laboratory of Ophthalmology, Shenzhen Eye Hospital, Jinan University, Shenzhen, China

**Keywords:** mitophagy, bevacizumab, anti-VEGF, retina, hypoxia, retinal neovascular disease

## Abstract

Anti-VEGF drugs are first-line treatments for retinal neovascular diseases, but these anti-angiogenic agents may also aggravate retinal damage by inducing hypoxia. Mitophagy can protect against hypoxia by maintaining mitochondrial quality, thereby sustaining metabolic homeostasis and reducing reactive oxygen species (ROS) generation. Here we report that the anti-VEGF agent bevacizumab upregulated the hypoxic cell marker HIF-1α in photoreceptors, Müller cells, and vascular endothelial cells of oxygen-induced retinopathy (OIR) model mice, as well as in hypoxic cultured 661W photoreceptors, MIO-MI Müller cells, and human vascular endothelial cells. Bevacizumab also increased expression of mitophagy-related proteins, and mitophagosome formation both *in vivo* and *in vitro*, but did not influence cellular ROS production or apoptosis rate. The HIF-1α inhibitor LW6 blocked mitophagy, augmented ROS production, and triggered apoptosis. Induction of HIF-1α and mitophagy were associated with upregulation of BCL2/adenovirus E1B 19-kDa protein-interacting protein 3 (BNIP3) and FUN14 domain containing 1 (FUNDC1), and overexpression of these proteins in culture reversed the effects of HIF-1α inhibition. These findings suggest that bevacizumab does induce retinal hypoxia, but that concomitant activation of the HIF-1α-BNIP3/FUNDC1 signaling pathway also induces mitophagy, which can mitigate the deleterious effects by reducing oxidative stress secondary. Promoting HIF-1α-BNIP3/FUNDC1-mediated mitophagy may enhance the safety of anti-VEGF therapy for retinal neovascular diseases and indicate new explanation and possible new target of the anti-VEGF therapy with suboptimal effect.

## Introduction

Retinal neovascularization (RNV) is a pathophysiological feature common to several retinal diseases, including retinopathy of prematurity (ROP), proliferative diabetic retinopathy (PDR), and retinal vein occlusion (RVO). ROP is the leading cause of blindness in childhood and DR is the leading cause in working-age adults. It is estimated that 230 million people worldwide are current living with RNV diseases, and prevalence is predicted to increase due to rising rates of diabetes (Fierson, 2018; Wong et al., 2018; Flaxel et al., 2020).

Vascular endothelial growth factor (VEGF) signaling is the predominant mechanism for pathological retinal neovascularization (Pierce et al., 1995; Campochiaro and Hackett, 2003; Campochiaro, 2015), so VEGF is the principal target of anti-angiogenic RNV treatments such as ranibizumab, aflibercept, and bevacizumab (Aiello et al., 1995; Campochiaro and Akhlaq, 2020). While these anti-VEGF drugs have shown promising efficacy in treating neovascularization of retinal tissue, many challenges remain. Numerous studies have demonstrated that VEGF is necessary for the survival of retinal and choroidal cells and that these pro-survival effects are independent of angiogenesis (Nishijima et al., 2007; Brockington et al., 2010). Accordingly, anti-VEGF drugs may compromise the *in vivo* requirement for VEGF and damage retina or choroidal cells. Indeed, approximately one-fifth of patients in the CATT trial developed geographic atrophy of the choriocapillaris and retinal pigmental epithelium (RPE) within 2 years after treatment (Grunwald et al., 2014), and Rofagha et al. (2013) reported that some treated eyes developed macular atrophy involving the foveal region. Studies investigating the direct ocular toxicity of anti-VEGF drugs by exposing cultured RPE and choroidal cells such as ARPE-19 cells to increasing concentrations of these compounds have not detected cellular apoptosis or toxicity within the therapeutic range (Malik et al., 2014). However, the pathogenic processes associated with RNV disease and treatment may be absent in culture systems consisting of a single cell type. For instance, cultured cell monolayers are usually well oxygenated. Consequently, further exploration is required to assess anti-VEGF toxicity in pathological contexts. In addition, a substantial proportion of patients do not respond optimally to these agents. In one trial, 41% of patients receiving aflibercept, 64% receiving bevacizumab, and 52% receiving ranibizumab for diabetic macular edema did not respond optimally and required focal/grid laser photocoagulation (Wells et al., 2016). These observations also suggest that effective therapy requires the suppression of pathological processes in addition to VEGF-A–VEGF receptor (VEGFR) signaling.

The hypoxia associated with anti-VEGF therapy may actually influence the clinical response. For instance, use of these agents for cancer chemotherapy increases tumor hypoxia *via* a hypoxia-inducible factor-1α (HIF-1α) signaling pathway (Miyazaki et al., 2014; Shi et al., 2017; Ueda et al., 2017; de Almeida et al., 2020). However, other studies have reported a decrease in tumor hypoxia (Lee et al., 2000; Dings et al., 2007; Myers et al., 2010). Further, the effects of anti-VEGF treatment for RNV diseases on retinal and cellular oxygenation status are largely uninvestigated.

Many hypoxic responses, including VEGF-dependent compensatory neovascularization, are mediated by a family of dimeric transcription factors termed hypoxia-inducible factors (HIFs). Cellular stressors including hypoxia also induce autophagy, a mechanism for degrading and recycling cellular components to maintain homeostasis (Manalo et al., 2005; Elvidge et al., 2006; Glick et al., 2010; Mazure and Pouysségur, 2010). Autophagy can be highly selective for specific cellular components and conditions (Manalo et al., 2005). For instance, selective autophagic degradation of damaged mitochondria inside lysosomes (termed mitophagy) is considered essential for the maintenance of metabolic homeostasis and the prevention of pathological processes initiated by mitochondrial dysfunction such as oxidative stress and apoptosis. Thus, excessive or insufficient mitophagy may lead to cell death, especially under stress (Wallace, 1999; Li et al., 2001; Galluzzi et al., 2012). Reactive oxygen species (ROS) are the byproduct of mitochondrial oxidative phosphorylation. ROS are generated at low levels by functional mitochondria and at an enhanced rate by damaged mitochondria and during reperfusion following hypoxia. Clearance of damaged mitochondria by mitophagy can prevent excessive ROS production and ensuing apoptotic cell death (Li et al., 2015). Mitophagy is regulated by both receptor-dependent and receptor-independent pathways. The BCL2 and adenovirus E1B 19kDa-interacting protein 3 (BNIP3) and FUN14 domain containing 1 (FUNDC1) are two important receptors in a recently described hypoxia-induced mitophagy pathway. These proteins localize on the outer mitochondrial membrane and interact with the autophagy-associated protein microtubule-associated protein 1 light chain 3 (LC3). Upon binding, an autophagic bilayer membrane envelope the mitochondrion to form an autophagosome, which then fuses with a lysosome to form an autophagolysosome within which the mitochondrion is degraded (Zhang et al., 2008; Liu et al., 2012; Ney, 2015).

Collectively, these findings suggest that anti-VEGF drugs may induce mitophagy *via* an HIF1-α pathway. However, potential effects on retina and therapeutic response are unknown. Here we examined the effects of the anti-VEGF drug bevacizumab on mitophagy in oxygen-induced retinopathy (OIR) model mouse retina as well as in cultured 661W photoreceptors, MIO-MI Müller cells, and human vascular endothelial cells (HUVECs) under hypoxia. We further investigate the functions of HIF-1α, BNIP3, and FUNDC1 on bevacizumab-induced changes in retinal cell oxidative stress, mitophagy, and survival.

## Materials and Methods

### Ethics Statement

Pregnant female wild type C57BL/6J mice were purchased from the Laboratory Animal Center of Southern Medical University (Guangzhou, China), and raised in the Experimental Animal Center of Zhongshan Ophthalmic Center, Sun Yat-sen University, under specific pathogen-free conditions. All procedures involving animals were conducted strictly in accordance with the Association for Research in Vision and Ophthalmology (ARVO) Statement for the use of Animals in Ophthalmic and Vision Research. All animal experiments were formally reviewed and approved by the Animal Care and Ethics Committee of the Zhongshan Ophthalmic Center (Approval number: 2020-023). All efforts were made to ensure the welfare and alleviate the suffering of animals.

### Cell Culture and Hypoxia Model

Human umbilical vein endothelial cells were obtained from the American Type Culture Collection (ATCC, #CRL-1730), while the 661W photoreceptor cell line was generously provided by Dr. Muayyad Al-Ubaidi (University of Oklahoma, Oklahoma City, OK, United States). The human Moorfield/Institute of Ophthalmology-Müller 1 (MIO-M1) cell line was established and characterized previously by Dr. Astrid Limb and colleagues (University College London, London, United Kingdom) (Limb et al., 2002). Cells were grown in Dulbecco’s Modified Eagle’s Medium F12 (DMEM/F12) supplemented with 10% fetal bovine serum (FBS) and 1% antibiotic mixture (penicillin and streptomycin) at 37°C in a humidified incubator under a 5% CO_2_ atmosphere. To mimic hypoxic conditions *in vitro*, cells were cultivated in serum-free DMEM in a portable three-gas controlled incubator (Smartor 118) under 5% CO_2_ and 1% O_2_ for 12 h at 37°C prior to harvesting.

### Transfections and Drug Treatments

BNIP3-overexpression plasmid and FUNDC1-overexpression plasmid were purchased from GeneCopoeia (United States). Briefly, human BNIP3 cDNA was cloned from NM_004052.4, mouse BNIP3 cDNA from NM_00976, human FUNDC1 from NM_173794, and mouse FUNDC1 cDNA from NM_028058. The resultant fragments were inserted into the OmickLink^TM^ Expression Vector pEZ-M02. Cultured 661W photoreceptors, Müller cells, and HUVECs were plated (1 × 10^6^ cell/6 well) in DMEM + FBS. The medium was then exchanged for Opti-MEM (Gibco) with Lipofectamine and 5 μg of the BNIP3- and FUNDC1-overexpression plasmids for 48 h using Lipofectamine 3000 (GLPBIO, United States, GK20006) under normal culture conditions. The HIF-1α inhibitor LW6 was purchased from (GLPBIO, United States, GC32724) and dissolved in dimethyl sulfoxide (DMSO) to a final concentration of 10 mM for cell application. Similarly, chloroquine (CQ) (GLPBIO, United States, G6423) was dissolved in DMSO to a final concentration of 10 mM. All these solutions were stored at −20°C before use. Subgroups of cells were incubated with 0.625 mg/mL bevacizumab (Avastin^®^, Roche, Switzerland), 0.5 mg/mL aflibercept (Eylea^®^, Bayer, Germany), or 0.125 mg/mL ranibizumab (Lucentis^®^, Novartis, Switzerland) as indicated. When combined with bevacizumab, LW6 and CQ were used at 25 μM.

### Western Blotting

Cells were harvested following treatment and cellular proteins isolated using standard procedures. Proteins were fractionated by SDS-PAGE and transferred to polyvinylidene difluoride membranes (Millipore, Billerica, MA, United States). Membranes were blocked with 5% skim milk for 60 min and then incubated with the following primary antibodies overnight at 4°C: anti-HIF-1α (1:1000, ABclonal, China, A11945), anti-LC3B (1:1000, Abcam, United Kingdom, ab48394), anti-BNIP3 (1:500, ABclonal, China, A5683), anti-FUNDC1 (1:500, ABclonal, China, A16318), anti-P62 (1:10000, Abcam, United Kingdom, ab109012), and anti-β-actin (1:10000, Bioss, China, bs-0061R). Blotted membranes were then incubated with 1:10000 horseradish peroxidase (HRP)-conjugated goat anti-rabbit secondary antibody (bs-0295G-HRP), and target bands visualized using an enhanced chemiluminescence detection system. Band intensities were calculated from gray scale images using ImageJ (Version 1.51). Target protein band intensities were normalized to β-actin band intensity (the gel-loading control). The ratio of cytosolic inactive LC3 (LC3-I) to lipidated LC3 (LC3-II) was calculated as an index of autophagic activation.

### Detection of Mitophagosome Formation by Laser Confocal Imaging

The Ad-HBmTur-Mito and Ad-LC3-GFP viral vectors (Hanbio, Shanghai, China) were used to directly detect mitophagosome formation by fluorescence confocal imaging. Briefly, cells were seeded on 35 mm glass-bottom dishes (NEST, China), allowed to be infected with virus for 8 h. The culture medium was then exchanged for fresh medium and cells incubated for 48–72 h before the indicated treatments (hypoxia and drug exposure). Treated cells were fixed with 4% paraformaldehyde and counterstained with 4, 6-diamidino-2-phenylindole dilactate (DAPI) (Solarbio, China, C0065). Confocal images of mitophagosomes were acquired using a confocal microscope (SP8 Leica, Germany).

### Immunofluorescence

Cells treated as indicated were fixed in 4% paraformaldehyde, permeabilized with 0.05% Triton X-100 for 15 min, blocked in 3% bovine serum albumin (BSA) for 1 h, then incubated overnight at 4°C with primary antibodies against BNIP3 (Abcam, United Kingdom, ab10433, 1:100) and FUNDC1 (Abcam, United Kingdom, ab224722, 1:100). Labeled cells were then incubated with Alexa Flour 594-conjugated goat secondary antibodies (1:200 BIOSS, China, bs-0295P-AF594 and bs-0296P-AF594) for 1 h at room temperature. Finally, cells were counterstained with DAPI, washed three times with PBS, and photographed using an inverted fluorescence microscope (Olympus FV1000, Olympus, Japan). At least three randomly chosen visual fields were photographed and analyzed for each sample. For double-stained retina tissue immunofluorescence, paraffin-embedded 4 μm sections were deparaffinized, dehydrated in gradient alcohol, heated in 20 min for antigen repair, then incubated with antibodies against LC3 (1:100, Abcam, United Kingdom, ab48394), HIF-1α (1:200, Abcam, United Kingdom, ab1), BNIP3 (1:100, Abcam, United Kingdom, ab10433), FUNDC1 (1:100, Abcam, United Kingdom, ab224722), CD31 (1:100, ABclonal, China, A4900), Rhodopsin (1:100, Abcam, United Kingdom, ab98887), and (or) Glutamine Synthetase (1:100, Abcam, United Kingdom, ab228590) overnight at 4°C. Immunolabeled retinal samples were incubated with Goat Anti-Rabbit IgG (HRP) (1:4000, Abcam, United Kingdom, ab205718) for 45 min and FITC-TSA-conjugated antibodies for 10 min. For incubation with the secondary primary antibody, sections were incubated in primary antibody rabbit overnight at 4°C followed by washing and incubation with Alexa Fluor^®^594 donkey anti-rabbit IgG (H + L) (A21207, Life Technologies, Germany). DAPI was used to counterstain cell nuclei. Images were captured using a confocal microscope (SP8, Leica, Germany).

### Cell Viability Assay

Cell viability was determined using a CCK-8 assay (GLPBIO, United States). This assay based on the conversion of the tetrazolium salt WST-8 by viable mitochondria to water-soluble WST-8 formazan. Briefly, cells were seeded in 96-well plates and treated as described. Cells were then incubated with 10 μL CCK-8 solution at 37°C for 2.5 h and the absorbance recorded at 450 nm as an estimate of viable cell number using a microplate reader (BioTek SynergyH1 microplate reader, United States).

### Measurement of Mitochondrial Membrane Potential (ΔΨm)

Changes in the mitochondrial membrane potential (ΔΨm) were measured by JC-1 staining (Solarbio, China). Cells were cultured on 35-mm glass-bottom dishes (NEST, China). Following the indicated treatments, cells were washed with PBS, incubated with 1 mL JC-1 working solution for 30 min at 37°C in the dark, and washed twice with JC-1 staining buffer.

The wavelength at excitation/emission 525/590 nm was used to assess JC-1 aggregates, and at excitation/emission 488/525 nm was used to assess JC-1 monomers under confocal microscopy (SP8, Leica, Germany) as an indicator of ΔΨm, with green emission indicative of relative depolarization.

### Measurement of Intracellular Reactive Oxygen Species

Intracellular ROS levels were measured using a fluorescent ROS Assay Kit (Solarbio, China). Cells were seeded in 6-well plates and incubated in 1 ml serum-free DMEM/F12 containing 1:1,000 dichloro-dihydro-fluorescein diacetate (DCFH-DA) for 20 min at 37°C under 5% CO_2_ as directed by the manufacturer. Cells were then washed three times in serum-free medium, deplated using trypsin, centrifuged at 600 × *g* for 4 min, and washed again with PBS. The fluorescence change due to ROS oxidation of DCFH-DA to DCFH was measured using a Fortessa X-20 flow cytometer (BD, United States) and analyzed using FlowJo (FlowJo, Ashland, OR, United States).

### TdT-Mediated dUTP Nick-End Labeling Staining for Apoptosis

Paraffin-embedded retinal sections were deparaffinized in dimethylbenzene, dehydrated in graded ethanol, incubated with proteinase K for antigen retrieval, washed three times in PBS, and then stained by using a TdT-mediated dUTP Nick-End Labeling (TUNEL) kit (Roche, Switzerland) followed by DAPI counterstaining of cell nuclei using an inverted fluorescence microscope (Olympus FV1000, Olympus, Japan).

### Oxygen-Induced Retinopathy Model

The C57/BL6J mice used to establish the OIR model were generated from pregnant females and reared by the Laboratory Animal Center of Southern Medical University (Guangzhou, China). The day of birth was defined as postnatal day (P0). At P7, both male and female neonatal mice and their nursing mothers were exposed to 75% oxygen for 5 days, followed by another 5 days of exposure to a normoxic environment prior to sacrifice at P17. Chloroquine (10 mg/kg) was given 1 h prior to bevacizumab vitreous injection and then every 24 h until sacrifice.

### Vitreous Injection

On P12, mice were anesthetized by 3% isoflurane followed by 2% isoflurane during drug injections. Eyes were first dilated with 1% tropicamide and treated with 0.5% proparacaine hydrochloride as a topical anesthetic (Alcaine, Alcon, United States). Mice then received intravitreal injection of bevacizumab (1 μL), LW6 (0.3 μL), or both (1 μL + 0.3 μL) under a stereomicroscope (Hamilton, United States) using a 33-gauge Hamilton syringe (Hamilton, United States).

### Detection of Tissue Hypoxia

To determine whether bevacizumab injection aggravates hypoxia in OIR model, the Hypoxyprobe Plus Kit (Hypoxyprobe Inc., Burlington, MA, United States) was used. 1 h before mice were euthanized, 60 mg pimonidazole hydrochloride per kg body weight was injected into the peritoneal cavities of mice. Retinal sections obtained from paraffin-embedded retinas were prepared as described above. Sets of sections were deparaffinized using xylene and then hydrated with decreasing concentrations of ethanol. Slides were then washed with PBS with 0.5% Tween 20, incubated for 10 min in 3% hydrogen peroxide at room temperature, and followed by another PBS with a 0.5% Tween 20 wash. Epitope unmasking was accomplished by incubating the sections in boiling 10 mM citrate buffer (pH 6.0) for 10 min. Anti-pimonidazole mouse monoclonal antibody, FITC-Mab1(1:50, Hypoxyprobe-1 Plus Kit, Hypoxyprobe Inc., Burlington, United States) was used as primary antibody with incubation overnight, and HRP linked to rabbit anti-FITC (1:50, Hypoxyprobe-1 Plus Kit, Hypoxyprobe Inc., Burlington, United States) was used as the secondary antibody for 30 min at room temperature according to the kit protocol. Immunohistochemical staining was performed with DAB (Chemicon International, United States, 71895) and were counterstained with hematoxylin (Solarbio, China, G1140). Images were acquired under Olympus FV1000 microscope (Olympus, Japan).

### Transmission Electron Microscopy

Eyes were excised at P17, fixed for 24 h in 4% glutaraldehyde, post-fixed in 1% osmium tetroxide, dehydrated in gradient ethanol, paraffin-embedded, and cut into 80 nm sections using an ultra-microtome. Sections were stained with uranyl acetate and lead citrate, followed by observation under transmission electron microscopy (TEM).

### Statistical Analysis

All data are expressed as mean ± SD and all statistical analyses were conducted using GraphPad Prism software (Version 5.0, United States). Group means were compared by one-way analysis of variance followed by *post hoc* Turkey’s tests for pair-wise comparisons. A *P* < 0.05 (two-tailed) was considered statistically significant for all tests.

## Results

### Bevacizumab Upregulated Hypoxia-Inducible Factor-1α Expression and Autophagic Markers in Cultured Photoreceptors, Müller Cells, and Vascular Endothelial Cells Under Hypoxia

**FIGURE 1:**
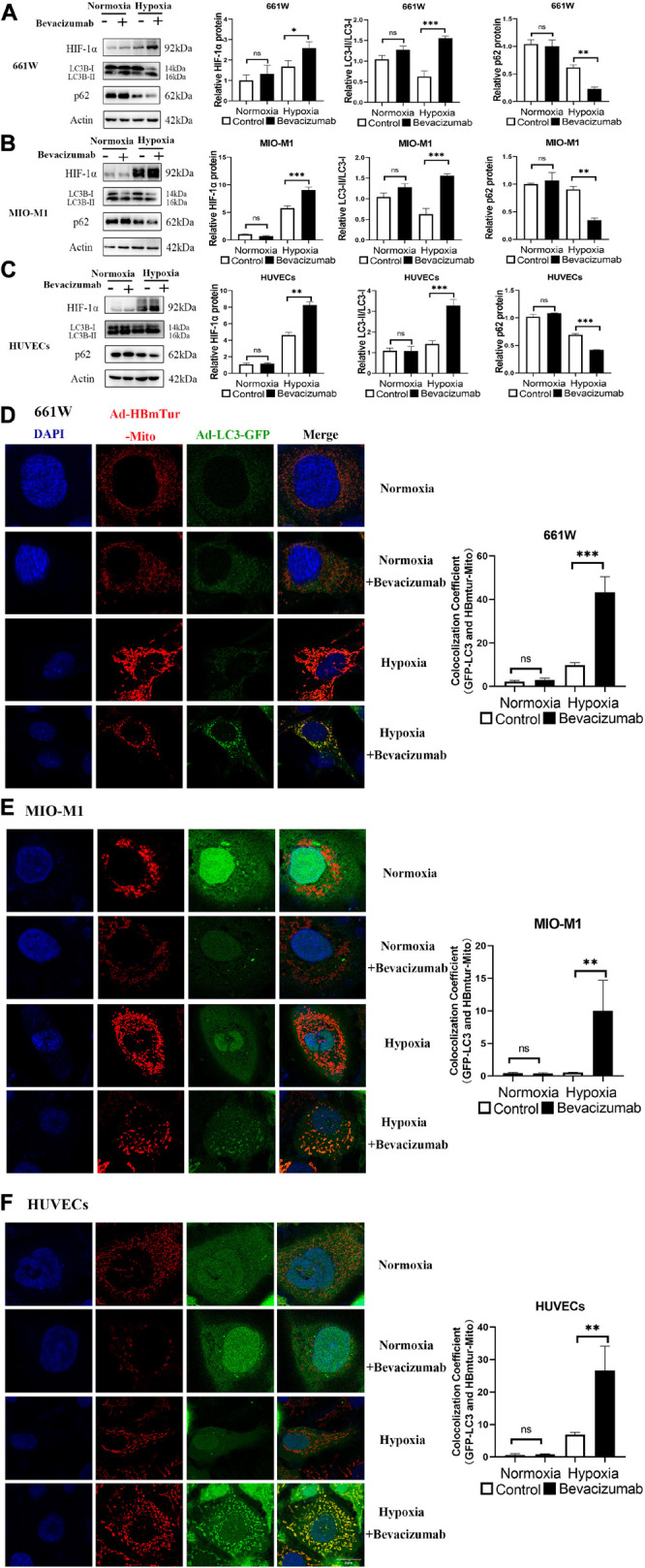
Bevacizumab exacerbated hypoxia and induces mitophagy in cultured photoreceptors, Müller cells, and vascular endothelial cells. **(A–C)** Representative western blots and densitometric results showing significant upregulation of HIF-1α and autophagy-related proteins LC3 and downregulation of P62 in hypoxic 661W photoreceptors, MIO-M1 Müller cells, and human umbilical vascular endothelial cells (HUVECs) following bevacizumab treatment compared with that in normoxia condition. (**D–F)** Representative immunofluorescence images and quantification showing that bevacizumab induced mitophagosome formation under hypoxia as indicated by LC3B and Mitotracker colocalization but no changes after bevacizumab treatment under normoxia. Bar: 10 μm. ns: no significance, ^∗^*P* < 0.05, ^∗∗^*P* < 0.01, ^∗∗∗^*P* < 0.001.

We examined whether bevacizumab increased HIF-1α and mitophagy in 661W cells, MIO-M1 cells, and HUVEC cells. HIF-1α was upregulated in all three cell types following bevacizumab treatment under 1% O_2_ hypoxia as measured by western blotting. Further, all cells exhibited an increased LC3-II/LC3-I ratio and downregulation of P62 (SQSTM1), consistent with induction of autophagy (Figures 1A–C). Moreover, cells transfected with Ad-GFP-LC3 and Ad-HBmTur-Mito showed enhanced colocalization of LC3 and Mitotracker following bevacizumab treatment under hypoxia condition but no changes after bevacizumab under normoxia condition, indicating an elevated rate of mitophagosome formation under hypoxia (Figures 1D–F).

### Bevacizumab Exacerbated Hypoxia and Increased Hypoxia-Inducible Factor-1α Expression and Autophagy in Photoreceptors, Müller Cells, and Vascular Endothelial Cells of Oxygen-Induced Retinopathy Model Retina

**FIGURE 2:**
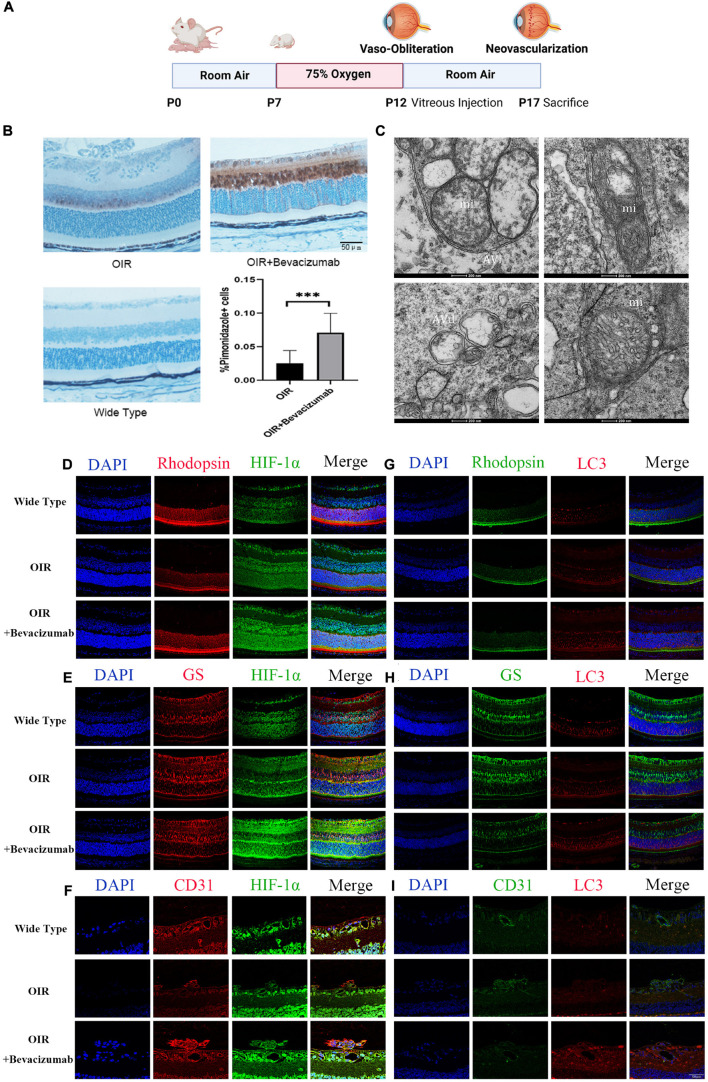
Bevacizumab exacerbated hypoxia and increased HIF-1α expression and autophagy in photoreceptors, Müller cells, and vascular endothelial cells of OIR model retina. **(A)** A time line schematic of oxygen-induced retinopathy (OIR) modeling. **(B)** Pimonidazole staining images and quantification showed more serious hypoxia within the retinal tissue after injected with bevacizumab in OIR mice. Bar = 50 μm. **(C)** Representative transmission electron microscopy (TEM) images depicting mitochondria, mitophagosomes, and mitolysosomes. In OIR mouse treated with bevacizumab, autophagosomes, referred as initial autophagic vacuoles (Avi) have double membrane contain mitochondria (mi) (upper left). Late and Degenerative autophagic vacuoles (Avd) have mitochondria at various stages of degradation (upper right and lower left), which have different morphology of mitochondria with normal mitochondria (lower right). Bar = 200 nm. **(D–F)** Representative dual immunofluorescence images showing HIF-1α accumulation in photoreceptors (rhodopsin-positive), Müller cells (glutamine synthetase-positive) and vascular endothelial cells (CD31-positive) following intravitreal injection of bevacizumab. Bar = 50 μm. **(G–I)** Representative dual immunofluorescence images showing LC3 upregulation in photoreceptors, Müller cells, and vascular endothelial cells following intravitreal injection of bevacizumab. Bar = 50 μm. ns: no significance, *P < 0.05, **P < 0.01, ***P < 0.001.

We then established an OIR model using the protocol described in Figure 2A. To explore whether bevacizumab exacerbates hypoxia in OIR model, we use Hypoxyprobe detected by immunohistochemistry to assess the hypoxic condition of retina. There is no positive staining in wide type mice retinal section which prove the specificity of the probe. The average color intensity of hypoxic areas (brown-stained retinal tissue) showed in retinas of the OIR mouse treated with bevacizumab is increased compared that in OIR model (Figure 2B). TEM was employed to further investigate whether mitophagy was occured in OIR combined with bevacizumab treatment. Mitophagy is a dynamic process and we get the static “snapshots” of different stage of mitophagy in OIR model injected with bevacizumab. In Figure 2C, compared with normal mitochondria in the retina of wide type mice (lower right), Upper left showed that mitochondria is engulfed by autophagosome at early stage. We observed that mitochondria degraded in upper right and normal morphology of mitochondria totally disappeared in lower left. Then we examined cell type-specific changes in oxygenation status induced by intravitreal bevacizumab injection by co-staining for HIF-1α and the photoreceptor marker rhodopsin, the Müller cell marker glutamine synthetase, or the vascular endothelial cells marker CD31 (Figures 2D–F). As shown in confocal images, HIF-1α immunoexpression was increased in all three cell types after intravitreal injection of 1 μL (per eye) 25 mg/mL bevacizumab (Avastin^®^). Similarly, co-staining for LC3-B and one of the aforementioned markers indicated increased autophagy in all three cell types (Figures 2G–I).

### Bevacizumab Upregulated the Mitophagy-Related Proteins BCL2/Adenovirus E1B 19-kDa Protein-Interacting Protein 3 and FUN14 Domain Containing 1

**FIGURE 3:**
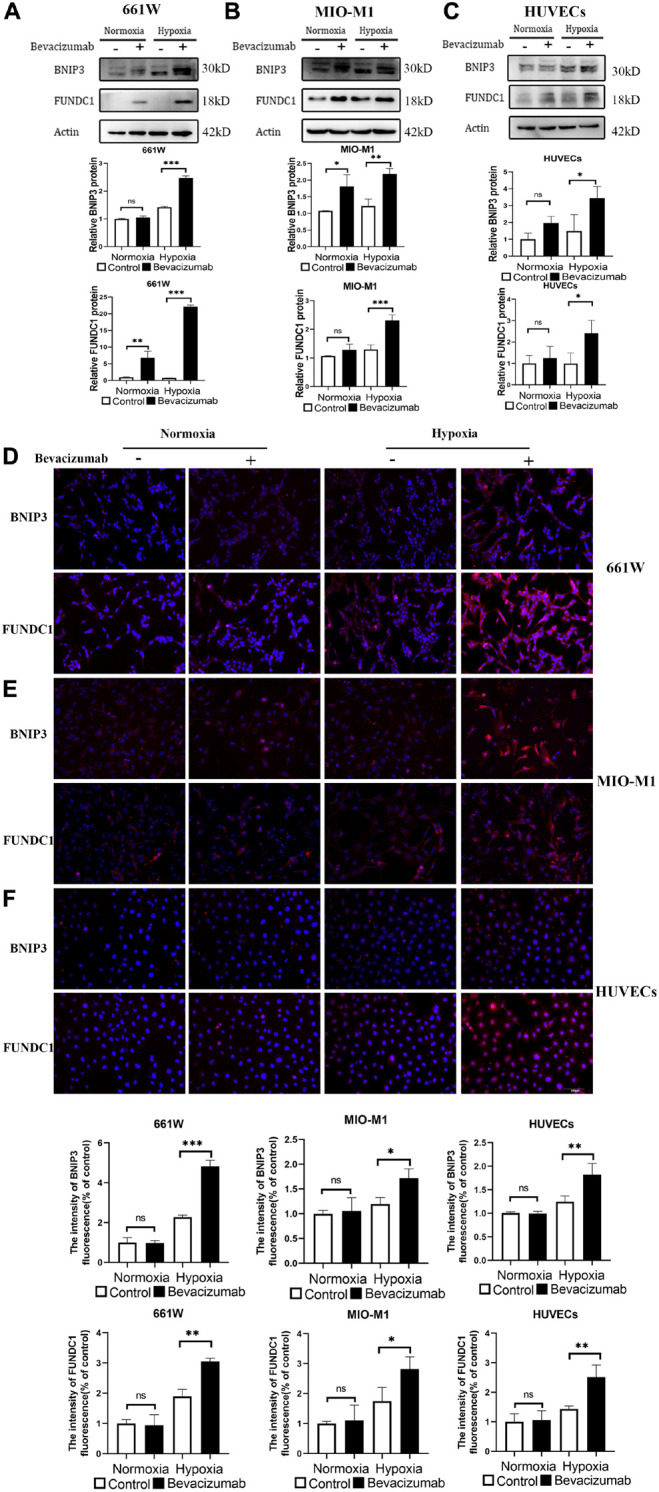
Mitophagy-related proteins BNIP3 and FUNDC1 were upregulated by bevacizumab treatment. **(A–C)** Representative western blot images and densitometric results showing upregulation of BNIP3 and FUNDC1 in 661W photoreceptors, MIO-M1 Müller cells, and HUVECs following bevacizumab treatment under hypoxia which was more significant compared to normoxia. **(D–F)** Representative immunofluorescence images and quantification confirming that bevacizumab upregulated BNIP3 and FUNDC1 in 661W photoreceptors, MIO-M1 Müller cells, and HUVECs especially under hypoxia. Bar: 100 μm. ns: no significance, ^∗^*P* < 0.05, ^∗∗^*P* < 0.01, ^∗∗∗^*P* < 0.001.

BCL2/adenovirus E1B 19-kDa protein-interacting protein 3 and FUNDC1 mediate ischemia-induced mitophagy by interacting with and recruiting LC3 to mitochondria. We examined whether BNIP3 and FUNDC1 participate in bevacizumab-induced mitophagy by measuring protein expression by western blot (Figures 3A–C) and immunofluorescence (Figures 3D–F). Both methods revealed that BNIP3 and FUNDC1 protein levels were significantly upregulated following bevacizumab treatment of cultured cells especially under hypoxia. These findings suggest that BNIP3 and FUNDC1 are potential downstream regulators of HIF-1α-mediated mitophagy.

### A Hypoxia-Inducible Factor-1α-BCL2/Adenovirus E1B 19-kDa Protein-Interacting Protein 3/FUN14 Domain Containing 1 Signaling Pathway Mediated Bevacizumab-Induced Mitophagy

**FIGURE 4:**
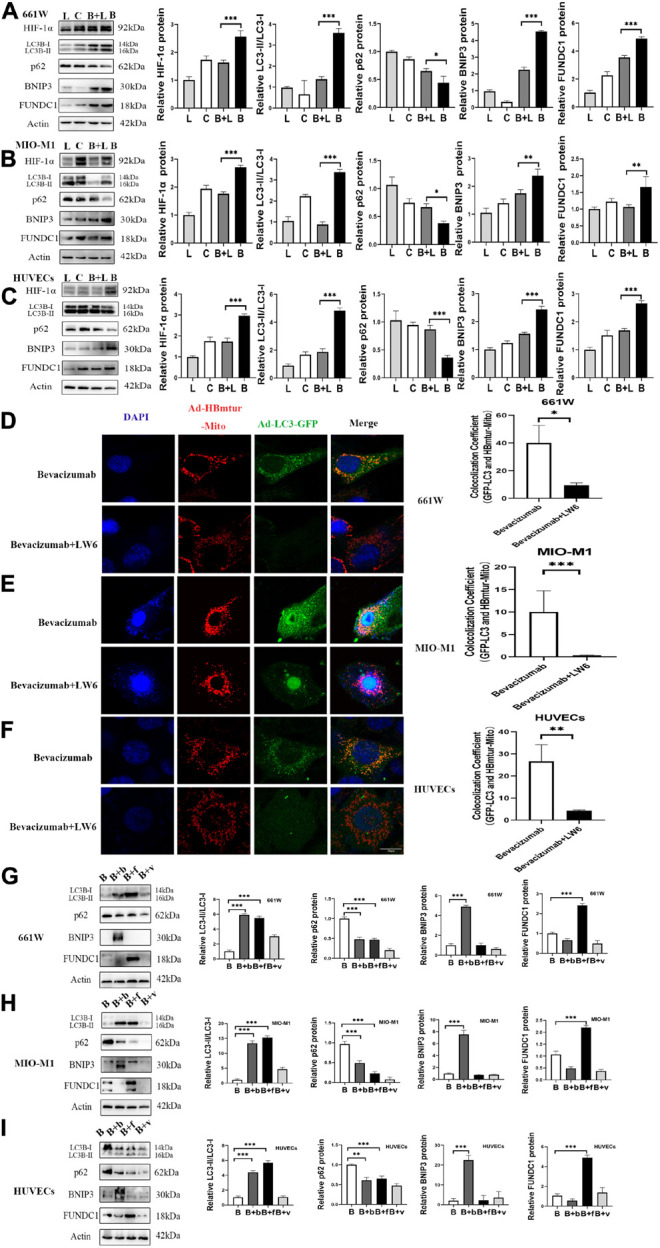
A HIF-1α-BNIP3/FUNDC1 signaling pathway mediated bevacizumab-induced mitophagy under hypoxia. **(A–C)** Representative western blot images and densitometric results showing that the upregulation of BNIP3, FUNDC1, and LC3-II/LC3-I ratio, and the downregulation of P62 following bevacizumab were reversed by the HIF-1α inhibitor LW6 under hypoxia. L, LW6; C, Control; B + L, Bevacizumab + LW6; B, Bevacizumab. **(D–F)** Representative immunofluorescence images and quantification showing that HIF-1α inhibition by LW6 decreased mitophagosome formation as indicated by reduced LC3B/Mitotracker colocalization under hypoxia. Bar: 10 μm. **(G–I)** Representative western blot images and densitometric results showing that transfection with BNIP3 or FUNDC1 overexpression plasmids reversed the inhibitory effect of LW6 on mitophagy-related protein expression. B: Bevacizumab; B + b: Bevacizumab + BNIP3 overexpression plasmid, B + f, Bevacizumab + FUNDC1 overexpression plasmid, B + v, Bevacizumab + plasmid vector. ns: no significance, ^∗^*P* < 0.05, ^∗∗^*P* < 0.01, ^∗∗∗^*P* < 0.001.

**FIGURE 5:**
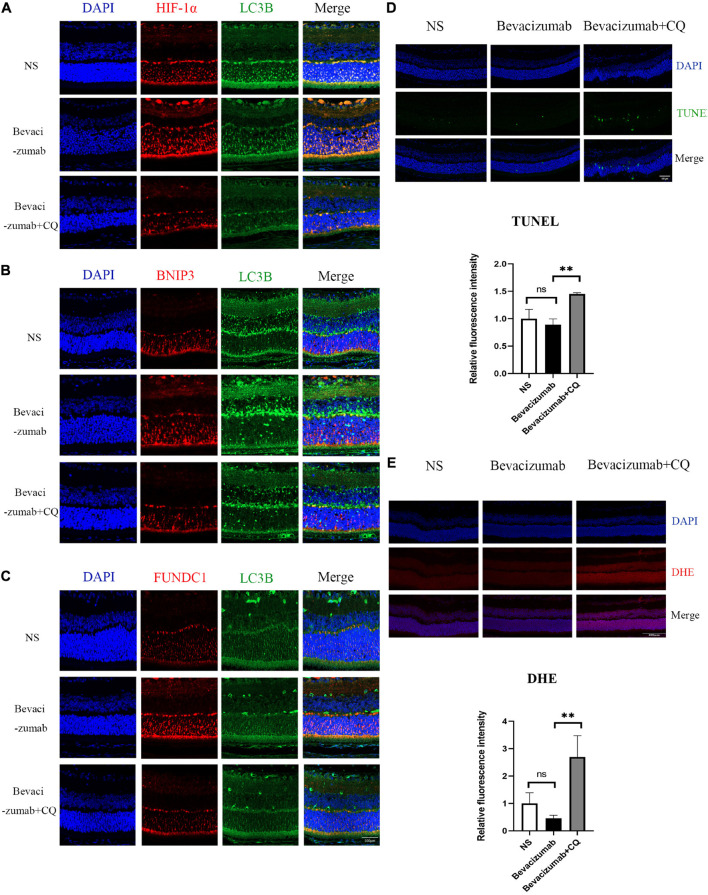
Hypoxia-inducible factor-BNIP3/FUNDC1-mediated mitophagy prevented bevacizumab-induced ROS accumulation and ROS-induced apoptosis in OIR mouse retina: **(A–C)** OIR mice received intravitreal injection of bevacizumab alone or bevacizumab plus LW6, Representative immunofluorescence images showing LC3 co-staining with HIF, BNIP3, or FUNDC1 in retina of OIR model. **(D)** OIR mice received intravitreal injection of bevacizumab alone or bevacizumab plus CQ injection, Representative TUNEL staining imaging that CQ suppressed mitophagy and promotes apoptosis in bevacizumab-treated retina. Bar = 100 μm. **(E)** Representative fluorescence images showing that CQ interfered with mitophagy and promotes ROS production in bevacizumab-treated retina. Bar:100 μm. ns: no significance, *P < 0.05, **P < 0.01, ***P < 0.001.

To examine whether bevacizumab-induced mitophagy induction is dependent on HIF-1α, BNIP3, and FUNDC1 upregulation, we measured protein expression levels in retinal cells co-treated with bevacizumab plus the HIF-1α inhibitor LW6 under hypoxia. As expected, HIF-1α expression was reduced by LW6. Consistent with a central role for HIF-1α/BNIP3/FUNDC1 signaling in bevacizumab-induced mitophagy, LW6 cotreatment also reduced the protein levels of BNIP3 and FUNDC1, decreased the LC3-II/LC3-I ratio, and upregulated P62 (Figures 4A–C). Further, the increased colocalization of Ad-GFP-LC3 and Ad-HBmTur-Mito following bevacizumab treatment under hypoxia was reversed by LW6 (Figures 4D–F). Therefore, inhibition of HIF-1α blocked both downstream BNIP3/FUNDC1 signaling and mitophagy. Conversely, transfection with BNIP3 and FUNDC1 overexpression plasmids reversed the inhibitory effect of LW6 on mitophagy-related proteins (Figures 4G–I). Moreover, co-injection of LW6 with bevacizumab reversed the elevations in LC3-B, HIF-1α, BNIP3, and FUNDC1 in OIR model (Figures 5A–C). These findings suggest that the HIF-1α-BNIP3/FUNDC1 signaling pathway mediates bevacizumab-induced mitophagy.

### Hypoxia-Inducible Factor-1α-BCL2/Adenovirus E1B 19-kDa Protein-Interacting Protein 3/FUN14 Domain Containing 1-Mediated Mitophagy Prevented Reactive Oxygen Species Accumulation and Reactive Oxygen Species-Induced Apoptosis Under Bevacizumab-Induced Hypoxia

**FIGURE 6:**
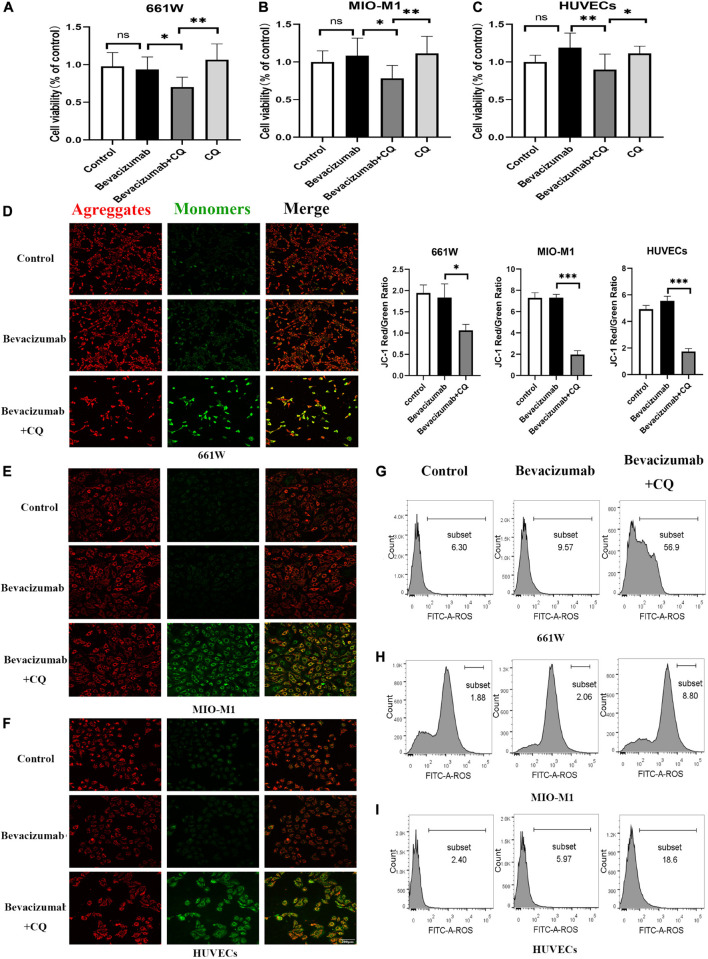
Hypoxia-inducible factor-1α-mediated mitophagy prevented bevacizumab-induced ROS accumulation and apoptosis *in vitro*: Cultured 661W photoreceptors, MIO-M1 Müller cells, and HUVECs were treated with bevacizumab or CQ alone, bevacizumab plus autophagy inhibitor CQ under hypoxia **(A–C)** cell viability was analyzed by CCK-8. (**D–F)** Mitochondrial membrane potential was analyzed by JC-1staing. Bar: 200 μm. **(G–I)** ROS was analyzed by DCFH-DA staining and flow cytometry. ns: no significance, ^∗^*P* < 0.05, ^∗∗^*P* < 0.01, ^∗∗∗^*P* < 0.001.

To examine if HIF-1α-BNIP3/FUNDC1-induced mitophagy can improve mitochondrial function under hypoxia, intracellular ROS production, cell viability, and apoptosis rate were measured in cultured hypoxic 661W, MIO-M1, and HUVEC cells were treated with bevacizumab or CQ alone or bevacizumab plus the mitophagy inhibitor CQ. The CCK-8 assay indicated that bevacizumab or CQ alone did not reduce the number of viable 661W photoreceptors, Müller cells, or HUVECs. However, addition of CQ did reduce the number of viable cells in the presence of bevacizumab, suggesting that HIF-1α mediated mitophagy promotes cell survival (Figures 6A–C). Cells stained with JC-1 emitted strong red fluorescence following vehicle or bevacizumab treatment, indicating well preserved ΔΨm polarization (a prerequisite for efficient oxidative phosphorylation), while cells stained with JC-1 and treated with bevacizumab in combination with CQ showed greater green fluorescence emission, which indicates ΔΨm depolarization (Figures 6D–F). Depolarization of the mitochondrial membrane is also a seminal early event in apoptosis, and TUNEL staining demonstrated that intravitreal injection of bevacizumab plus intraperitoneal CQ injection enhanced apoptotic cell numbers compared to bevacizumab injection only (Figure 5D). Further, inhibition of mitophagy increased intracellular ROS levels in cultured cells during bevacizumab treatment compared to bevacizumab treatment alone as measured by DCFH-DA staining and flow cytometry (Figures 6G–I). Similar results were obtained in OIR mice. Intravitreal injection of bevacizumab plus CQ injection enhanced ROS production, while bevacizumab injection alone had no detectable effect on intracellular ROS (Figure 5E). Thus, despite exacerbation of hypoxia, bevacizumab did not induce oxidative stress or apoptosis if HIF-1α-mediated mitophagy was maintained, suggesting that HIF-1α-mediated mitophagy normally serves to protect retinal cells against hypoxic damage from bevacizumab.

### Other Anti-Vascular Endothelial Growth Factor Agents Have Similar Effects on Cell Viability

**FIGURE 7:**
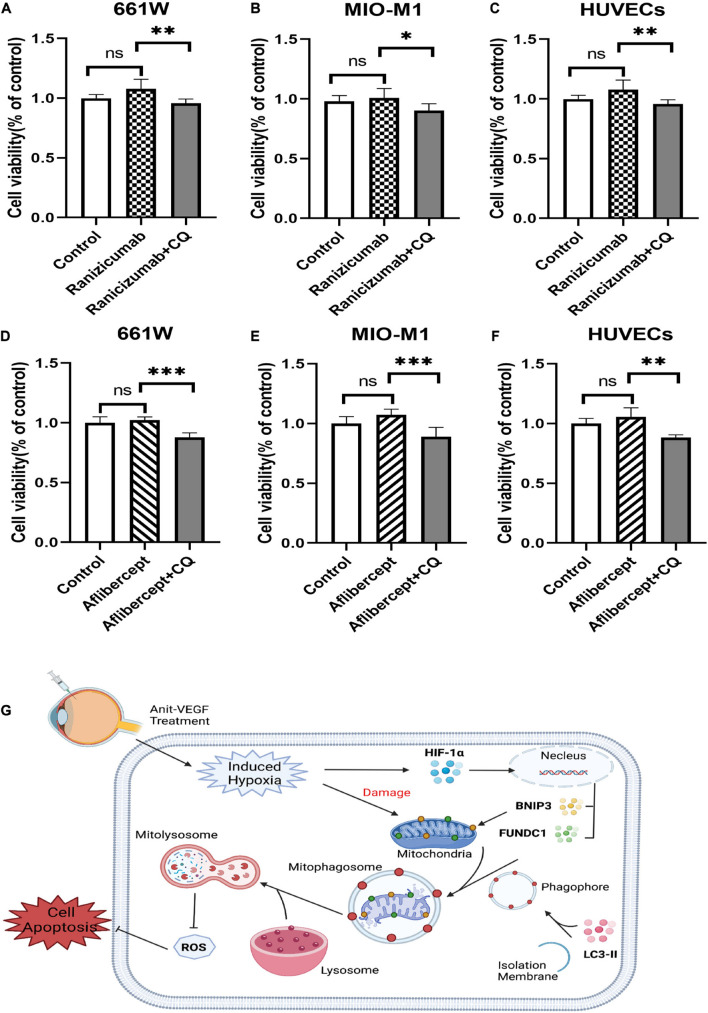
Ranibizumab and aflibercept had same effects with bevacizumab on cell viability in cells of retina. 661W photoreceptors, MIO-M1 Müller cells, and HUVECs were treated with ranibizumab and aflibercept alone or in combination with CQ. **(A–F)** Cell viability was analyzed by CCK-8 assay. **(G)** Schematic diagram illustrating that anti-VEGF treatment can exacerbate hypoxia but also induce HIF-1α/cBNIP3/FUNDC1-mediated mitophagy to reduce ROS production and apoptosis, thereby maintaining cell viability. ns: no significance, *P < 0.05, **P < 0.01, ***P < 0.001.

Several anti-VEGF agents have been approved for therapeutic use, so we also investigated the effects of two additional drugs, ranibizumab (Lucentis^®^), and aflibercept (Eylea^®^). Neither agent induced measurable cytotoxicity when applied alone to hypoxic cultured retinal cells as indicated by CCK-8 assay. However, cell viability was reduced by addition of mitophagy inhibitors (Figures 7A–F). Thus, HIF-1α-dependent mitophagy is broadly protective against retinal damage from anti-VEGF drug-induced hypoxia. These findings are summarized by a schematic diagram (Figure 7G). Anti-VEGF treatment can exacerbate retinal hypoxia but also induces HIF-1α/BNIP3/FUNDC1-mediated mitophagy, which sustains mitochondrial function and thereby reduces oxidative stress and apoptosis (Figure 7G).

## Discussion

In this study, we examined if the efficacy and safety of anti-VEGF drugs for RNV diseases are limited by induction of hypoxia. While intravitreal bevacizumab did indeed exacerbate hypoxia in the mouse retina and enhance the hypoxic responses of cultured retinal cells, we also found that these responses were accompanied by elevated mitophagy and that this concomitant mitophagy acted to preserve mitochondrial function and reduce apoptosis. Further investigations showed that mitophagy induction was dependent on activation of the HIF-1α-BNIP3/FUNDC1 signaling pathway and that inhibition of HIF-1α and mitophagy increased oxidative stress and apoptosis in response to bevacizumab. Thus, mitophagy acts as a vital protective mechanism during bevacizumab treatment.

Expression of HIF-1α was increased in photoreceptors, Müller cells, and vascular endothelia cells exposed to bevacizumab as evidenced by western blotting of lysates from cultured 661W photoreceptors, MIO-M1 Müller cells, and HUVECs. It was noteworthy that these changes were not detected under normoxia but was notably induced by hypoxia. These findings were further verified by co-immunostaining for HIF-1α and cell-specific markers in OIR model, an *in vivo* model of hypoxia-induced ocular neovascularization. Increased levels of HIF-1α can indirectly reflect enhanced hypoxia exposure in bevacizumab treated cells and tissue in retina. We then use Hypoxyprobe to provide direct support for this. Pimonidazole hydrochloride (HP-1) is reductively activated in hypoxic tissue and forms stable adducts with thiol (sulphydryl) groups in proteins, peptides and amino acids. The images showed more serious hypoxia within the retinal tissue after injected with bevacizumab in OIR mice. Thus, bevacizumab appears to enhance retinal hypoxia, in according with previous suggestions that bevacizumab may induce local retinal hypoxia and alter photoreceptor function (Schraermeyer and Julien, 2012). Hypoxia is a critical driver of pathological neovascularization, and HIF-1α is a hypoxia-sensing transcription factor controlling the expression of hypoxia-inducible genes. Thus, bevacizumab may amplify the responses of retinal cells to hypoxia. Similarly, several studies have reported that anti-VEGF therapy increased tumor hypoxia. Neovascularization is a compensatory process meant to restore oxygen homeostasis and promote recovery from hypoxic damage, and blocking the formation of new blood vessels may increase intra-tumor hypoxia, which is the rationale for anti-VEGF therapy in cancer patients. Meyer et al. (2009) also found that bevacizumab could activate platelet FcRIIa in monkeys, resulting in platelet aggregation, degranulation, and thrombosis. Thus, bevacizumab may promote tissue hypoxia through multiple pathways.

Transcription factor HIF-1α is implicated in the regulation of many physiological and pathological processes, including angiogenesis, apoptosis, proteolysis, metabolism, cell survival, cell migration, and tumor invasion (Dayan et al., 2006; Pouysségur et al., 2006) as well as autophagy (Tracy et al., 2007). Autophagy or “self-eating” by lysosomes was first observed in the 1950s (Yang and Klionsky, 2010) and is now considered an ubiquitous stress response that facilitates the removal of dysfunctional or superfluous cellular components and the recycling of base elements for both biosynthesis and energy generation (Levine and Kroemer, 2019). Autophagy has long been considered a non-selective process but it is now known that some organelles, such as mitochondria and endoplasmic reticulum, can be selectively targeted by the autophagic machinery (Zaffagnini and Martens, 2016). Mitochondria are the main sites of aerobic respiration and so are highly sensitive to cellular oxygen levels. If oxygen levels become unstable, mitochondrial ATP generation may be reduced and ROS generation increased, which can induce local damage. Under these conditions, mitophagy may mitigate oxidative stress and downstream pathology by removing damaged mitochondria (Kim et al., 2007), while insufficient or excessive mitophagy could lead to further damage and cell death. In our models, mitophagy appeared to protect against hypoxia induced by anti-VEGF treatment (Shintani and Klionsky, 2004). Cell viability, mitochondrial membrane potential, and apoptosis assays indicated that bevacizumab had little deleterious effect on retinal cells, even under hypoxia. However, inhibition of mitophagy reduced cell viability, depolarized the mitochondrial membrane potential, and increased apoptosis rate.

We then investigated the mechanisms linking bevacizumab treatment to mitophagy and mitophagy to cellular protection. Reactive oxygen species (ROS), including superoxide, hydroxyl radical, nitric oxide, hydrogen peroxide, and singlet oxygen, are generated directly by the mitochondrial electron transport chain driving oxidative phosphorylation and by various downstream reactions. Damaged mitochondria release more ROS (Oyewole and Birch-Machin, 2015), while mitophagy may prevent ROS generation and ensuing oxidative stress by removing damaged mitochondria (Wang, 2001; Dan Dunn et al., 2015). Indeed, inhibition of HIF-1α- mediated mitophagy resulted in higher ROS generation during bevacizumab exposure, suggesting that mitophagy is necessary for the maintenance of mitochondrial quality. Mitophagy is classically mediated by the PINK-PARKIN pathway (Kitada et al., 1998) but several additional receptor-mediated mitophagy pathways have been described. Mitophagy receptors such as Atg32, BNIP3, and FUNDC1 are localized to the outer mitochondrial membrane and have the classic tetrapeptide sequence allowing interactions with LC3 (Liu et al., 2014). In addition to changes in mitochondrial function, hypoxia is known to trigger autophagy (Bursch et al., 2008) and several independent studies have demonstrated that BNIP3 and FUNDC1 are necessary for induction (Chourasia et al., 2015; O’Sullivan et al., 2015; Li et al., 2019; Livingston et al., 2019). BNIP3 was first identified as a Bcl-2-interacting molecule that regulates apoptotic death and programmed necrosis but also contains a typical LIR motif for interaction with the autophagy-targeting protein LC3. FUN14 domain-containing 1 (FUNDC1), a three transmembrane domain protein localized on the outer mitochondrial membrane with an N-terminus domain exposed to the cytosol, also processes a LIR motif allowing interaction with LC3 (Hanna et al., 2012). Bevacizumab treatment upregulated BNIP3 and FUNDC1 in addition to HIF-1α, while pharmacological inhibition of HIF-1α reversed BNIP3 and FUNDC1 upregulation and concomitantly decreased the expression of other mitophagy-related proteins. Conversely, BNIP3 or FUNDC1 overexpression plasmids reversed the downregulation of mitophagy-related proteins by HIF-1α inhibition. These findings strongly suggest that HIF-1α induced mitophagy *via* receptors BNIP3 and FUNDC1.

In contrast to previous studies on the cellular effects of anti-VEGF drugs, we examined *in vitro* cellular responses under hypoxia to better approximate the clinical condition and combined culture experiments with examination of OIR mice, a well-accepted *in vivo* angiogenesis model. Müller cells are the principal glial cell type in retina and provide essential structural support as well as trophic and metabolic support to neurons and photoreceptors. In addition, Müller cells protect neurons from exposure to excess neurotransmitters by regulating the extracellular concentration through uptake mechanisms (Reichenbach and Bringmann, 2013). Photoreceptors are the primary sites of light transduction and provide primary outputs to bipolar and horizontal cells, which in turn active ganglion cells, the primary output cells. Photoreceptors have high metabolic demands that thus require efficient delivery of nutrients and oxygen from retinal vessels (Lamb, 2013). Therefore, it is expecting that cellular functions would be markedly disrupted by anti-VEGF drug-induced hypoxia. However, bevacizumab had no deleterious effects on photoreceptors or Müller cells under hypoxia but did induce oxidative stress and apoptosis under mitophagy inhibition. Inhibition of mitophagy also suppressed HUVECs viability. Thus, mitophagy in HUVECs is an adaptive response that hampers the efficacy of anti-VEGF drugs to suppress angiogenesis. Strategies to inhibit mitophagy in vascular endothelial cells while promoting mitophagy in photoreceptors, Müller cells, and neurons may therefore optimize therapeutic efficacy and safety.

Finally, we also report that two additional anti-VEGF drugs, ranibizumab and aflibercept, did not influence cell viability under hypoxia conditions, while inhibition of mitophagy reduced cell viability. Thus, concomitant mitophagy appears to be a common protective mechanism under anti-VEGF treatment.

In summary, the present study demonstrates that photoreceptors, Müller cells, and vascular endothelial cells are not adversely affected by anti-VEGF agents even under hypoxia due to concomitant activation of HIF-1α-BNIP3/FUNDC1-mitophagy. The regulation of mitophagy in specific retinal cell types may be a useful strategy to optimize the therapeutic efficacy and safety of anti-VEGF drugs.

## Data Availability Statement

The original contributions presented in the study are included in the article/supplementary material, further inquiries can be directed to the corresponding author/s.

## Ethics Statement

The animal study was reviewed and approved by Animal Care and Ethics Committee of the Zhongshan Ophthalmic Center (Approval number: 2020-023).

## Author Contributions

SZ, WC and YS contributed to the study concept and design. YS, LS, and JL contributed to the experimental and technical support. YS and CY contributed to the acquisition, analysis, or interpretation of data. WC and YS contributed to the drafting of the manuscript and statistical analysis. WC, FW, and YS contributed to the critical revision of the manuscript for important intellectual content. All authors contributed to the article and approved the submitted version.

## Conflict of Interest

The authors declare that the research was conducted in the absence of any commercial or financial relationships that could be construed as a potential conflict of interest.

## Publisher’s Note

All claims expressed in this article are solely those of the authors and do not necessarily represent those of their affiliated organizations, or those of the publisher, the editors and the reviewers. Any product that may be evaluated in this article, or claim that may be made by its manufacturer, is not guaranteed or endorsed by the publisher.
